# Not an ‘either/or’: Integrating mental health and psychosocial support within non-communicable disease prevention and care in humanitarian response

**DOI:** 10.7189/jogh.11.03119

**Published:** 2021-11-13

**Authors:** Bishal Gyawali, Mary C Harasym, Saria Hassan, Katy Cooper, Anouk Boschma, Martha Bird, Flemming Konradsen, Emmanuel Raju, Siri Tellier

**Affiliations:** 1 Section of Global Health, Department of Public Health, University of Copenhagen, Copenhagen, Denmark; 2 IFRC Reference Centre for Psychosocial Support, Copenhagen, Denmark; 3 Emory School of Medicine, Atlanta, Georgia, USA; 4 Copenhagen Centre for Disaster Research, University of Copenhagen, Denmark

Noncommunicable diseases (NCDs) – including cardiovascular diseases, cancers, diabetes, mental ill-health and chronic respiratory diseases – are highly prevalent in humanitarian settings. In 2017, NCDs accounted for 24 to 68 percent of the mortality in migrants from the most common countries of migrant origin, consisting of Syria, Afghanistan, South Sudan, Myanmar, and Somalia [1]. About one-fifth of those living in humanitarian settings suffer from mental ill-health, such as depression, anxiety and posttraumatic stress disorder [2].

Mental ill-health and physical NCDs are often linked by the underlying individual, community, and societal factors, and frequently co-occur with interdependent causation [3]. For instance, physical NCDs can lead to depression and anxiety, whereas mental ill-health can result in decreased help-seeking, poor treatment adherence and poorer prognoses for physical NCDs. Most of the disease burden in humanitarian settings is attributable to these multi-faceted and often co-morbid conditions, and short-term, disease-specific responses fail adequately to prioritize and address these conditions [4].

Although people in humanitarian response demonstrate significant resilience that draws on social and individual protective factors, experiences in humanitarian response can contribute to significant mental health and psychosocial difficulties, leading to the need for effective interventions, such as mental health and psychosocial support (MHPSS) services. These services range from 1) the promotion of positive mental health and well-being through psychological first aid and recreational activities, to 2) prevention activities via peer support groups, families, and individuals, to 3) basic psychological interventions, such as counselling and psychotherapy, which are usually provided in health care facilities with accompanying outreach work in community facilities, to specialized clinical care and treatment for individuals with chronic mental ill-health and for persons suffering such severe distress and over such a period that they have difficulties coping in their daily lives, such as activities in treatment centres for survivors of torture and alternative approaches to drug therapy [5].

The existing guidelines for international humanitarian aid have now emphasized the need to address NCDs through investing in MHPSS efforts that improve health outcomes and reduce morbidity [4,6]. However, it is only recently that NCD prevention and care have started to receive attention within humanitarian settings, and little is being done to take advantage of synergies by integrating MHPSS within NCD prevention and care. Furthermore, there is a lack of operational standardization due to the limited availability of an evidence base to inform the guidance and response, and the failure to include specifically community-based PSS considerations [7].

A particular challenge to integration is that the health systems operate in silos – with separate guidelines for care and for providers trained to deliver specific, rather than integrated, forms of care. Too often, health care facilities operate with vertical models, with NCD care and MHPSS managed separately in clinical practice and primary care [8]. This separation also exists in upstreaming in the financing of service delivery. Most health care facilities in humanitarian settings receive funding from a wide range of bilateral, multilateral, and private sources. Most donor funds, however, finance vertical and segmented programmes based on requirements, target populations, priorities, and outcomes, and tend to be short-term and targeted [8]. As a result, services become fragmented, redundant, inefficient, and time-consuming, hampering integration by limiting time and continuity of care [9]. More recently, the COVID-19 pandemic has exposed how deeply entwined mental ill-health and other NCDs can be and the perils of continuing to treat these conditions in silos, especially in countries that are experiencing humanitarian crises with limited resources to tackle both fighting pandemics and treating existing illnesses [10]. The indirect impacts of the pandemic (such as the lockdowns) have negatively impacted the management of NCDs and mental ill-health, including a lack of support and access to facilities for improving lifestyle management, monitoring patients, and providing essential medicines on a regular basis [11,12].

Given the scarcity of resources and competition from vertical and segmented project funding among donors in humanitarian settings, there is a need to redesign systems that leverage scarce resources to better meet populations' needs holistically. Where possible, MHPSS should be included as an integral part of the community- and primary-level NCD services in humanitarian settings, taking a tiered approach that is responsive to individual needs (from basic MHPSS support for all to PSS or peer-support groups for vulnerable populations, and individual counselling and treatment where appropriate). Such integration can be bidirectional, with multi-level and multi-sectoral MHPSS approaches being incorporated into NCD prevention and care, or vice versa with NCD prevention and care approaches being integrated into MHPSS services.

**Figure:**
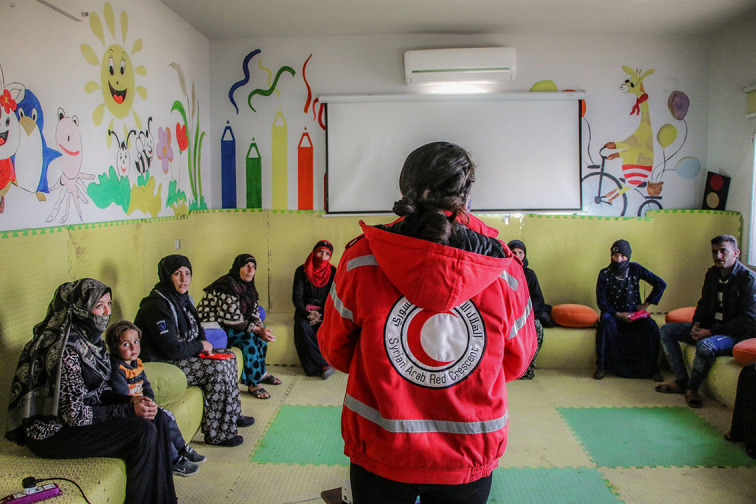
Photo: Syrian refugees attending mental health and psychosocial support programme in the city of Swada in southwestern Syria. A two-month programme helped them learn parenting and vocational skills as well as general life skills and ways to cope with different situations. From: PS Centre Comms.

Despite mental health being prioritized in humanitarian settings in recent years [5], limited human resources make it difficult to operationalize frameworks that facilitate effective integration of MHPSS into NCD services and vice versa. A shortage of practitioners and the emphasis on specialists (psychologists, psychiatrists) over generalists (lay health workers) are some of the barriers in humanitarian settings [13]. Specialists are essential for mental health care, but the training of general practitioners and lay health workers, as well as strengthening family and community supports, and encouraging self-help, are equally important. Redistributing care tasks from more specialized clinically highly qualified health care workers to those with less formal, less specialized and less clinical training and qualifications but with more supervision, ideally from both specialists and peers and sharing tasks with an equally qualified and/or the multidisciplinary cadre of health care workers (such as physicians, psychologists, nurses and social workers) can allow for greater reach of MHPSS services and more efficient use of human resources.

Finally, socio-cultural barriers, such as discrimination and stigma, pose a significant barrier to MHPSS and NCD care integration [14]. Significant stigma and discrimination against people living with NCDs, particularly mental ill-health, may be rooted in cultural norms, and in many places, mental health support has been provided through sources of help such as family and religious leaders rather than through the formal health care system in humanitarian response. Consequently, it is found to affect both the acceptance of mental ill-health and the willingness of the patient or affected person to seek care. A study conducted among Syrian refugees living with NCDs in Jordan reported that although patients recognised the psychological dimensions of their illness, they were reluctant to attend clinic-based MHPSS services due to the fear of discrimination [15]. Moreover, the study reported that doctors tended to be reluctant to refer patients for MHPSS, as they were concerned about stigmatization [15].

MHPSS-focused awareness and sensitization through social media, peer counselling and community and outreach involvement in providing psycho-education and mobile MHPSS services can help reduce stigma and discrimination among patients, enable better adherence and access to treatment, and promote help-seeking behaviours at both the individual and community levels [16]. Healthcare workers, especially those in primary care, and religious leaders and faith healers, as well as community leaders, should be considered the important target group for anti-stigma interventions, which are important in improving mental health literacy, attitude, and understanding of MHPSS.

Greater engagement with NCD patients and the provision of patient-centered care would foster the co-creation of culturally appropriate programmes that better meet the population’s physical NCD needs and better anticipate their context-specific MPHSS needs. This cultural appropriateness should include consideration of the stigma attached to such support. This could also be reinforced by situating the programmes in non-clinical settings, such as community halls or schools, to increase both their acceptability and accessibility.

Thus, advancement in advocacy, research, policy, and practice is urgently needed to integrate MHPSS and NCD prevention and care in humanitarian response. We urge humanitarian actors and policymakers in collaboration with researchers to:

- recognise the benefits of integration of MHPSS and NCD prevention and care in humanitarian response;
- increase investment in operational research to develop, strengthen and evaluate innovative, effective implementation strategies to integrate MHPSS in NCD prevention and care;
- produce guidelines for implementation of effective integration that build upon practice-based evidence towards evidence-informed and evidence-based practice for effective integration;
- advocate with larger global, regional and national stakeholders, raise awareness and increase outreach efforts with local and community stakeholders to reduce stigma about seeking care for NCDs, including mental ill-health; and
- devise sustainable humanitarian financing mechanisms for integration.
